# Added Value of Next-Generation Sequencing for Multilocus Sequence Typing Analysis of a *Pneumocystis jirovecii* Pneumonia Outbreak^1^

**DOI:** 10.3201/eid2308.161295

**Published:** 2017-08

**Authors:** Elena Charpentier, Cécile Garnaud, Claire Wintenberger, Sébastien Bailly, Jean-Benjamin Murat, John Rendu, Patricia Pavese, Thibault Drouet, Caroline Augier, Paolo Malvezzi, Anne Thiébaut-Bertrand, Marie-Reine Mallaret, Olivier Epaulard, Muriel Cornet, Sylvie Larrat, Danièle Maubon

**Affiliations:** Centre Hospitalier Universitaire Grenoble Alpes, Grenoble, France (E. Charpentier, C. Garnaud, C. Wintenberger, J.-B. Murat, J. Rendu, P. Pavese, T. Drouet, C. Augier, P. Malvezzi, A. Thiébaut-Bertrand, M.-R. Mallaret, O. Epaulard, M. Cornet, S. Larrat, D. Maubon);; Université Grenoble Alpes, Grenoble (E. Charpentier, C. Garnaud, M.-R. Mallaret, O. Epaulard, M. Cornet, S. Larrat, D. Maubon);; Institut National de la Santé et de la Recherche Médicale Université Paris Diderot, Paris, France (S. Bailly).

**Keywords:** Pneumocystis jirovecii, pneumocystosis, pneumonia, disease outbreaks, high-throughput nucleotide sequencing, transplants, multilocus sequence typing, next-generation sequencing, NGS, MLST, respiratory infections, fungi

## Abstract

*Pneumocystis jirovecii* is a major threat for immunocompromised patients, and clusters of pneumocystis pneumonia (PCP) have been increasingly described in transplant units during the past decade. Exploring an outbreak transmission network requires complementary spatiotemporal and strain-typing approaches. We analyzed a PCP outbreak and demonstrated the added value of next-generation sequencing (NGS) for the multilocus sequence typing (MLST) study of *P. jirovecii* strains. Thirty-two PCP patients were included. Among the 12 solid organ transplant patients, 5 shared a major and unique genotype that was also found as a minor strain in a sixth patient. A transmission map analysis strengthened the suspicion of nosocomial acquisition of this strain for the 6 patients. NGS-MLST enables accurate determination of subpopulation, which allowed excluding other patients from the transmission network. NGS-MLST genotyping approach was essential to deciphering this outbreak. This innovative approach brings new insights for future epidemiologic studies on this uncultivable opportunistic fungus.

*Pneumocystis jirovecii* pneumonia (PCP) is a life-threatening opportunistic disease that poses a particular threat in healthcare units that manage patients with oncohematologic conditions, patients having received solid organ transplants (SOTs), and HIV-infected patients. The incidence of PCP among SOT recipients has increased markedly since the early 2000s, concomitant with the development of more intensive immunosuppressive therapy and increasing organ transplant procedures (*1*). During 2001–2010, the incidence of PCP among HIV-positive/AIDS patients in France dropped from 2.4 to 0.7 cases/10^5^/year (−14.2%/year), whereas incidence increased from 0.13 to 0.35 cases/10^5^/year (+13.3%/year) among patients without HIV infection (*2*). For SOT patients in France, the incidence of PCP increased by 13% during 2004–2010 (*2*). Moreover, multiple outbreak clusters in this specific population have been reported (*3*–*5*). Patients receiving renal transplants are particularly at risk; most reported PCP clusters have occurred in kidney transplant units (*6*). These outbreaks support the hypothesis of a de novo person-to-person transmission of *P. jirovecii* and its potential for nosocomial spread (*7*).

Exploring an outbreak transmission network requires spatiotemporal and strain-typing approaches. Various genotyping approaches to the study of *P. jirovecii* epidemiology have been described (*8*–*12*). Genotyping based on multilocus sequence typing (MLST) is standard for strain characterization, especially for organisms that are not easily cultivable, because it provides excellent discriminatory power and reproducibility (*3*,*4*,*13*,*14*). Multiple *P. jirovecii* strains isolated from a unique respiratory sample have frequently been reported (*6**–**8**,**10*). The proportions of these mixed strains in samples varied substantially according to the genotyping method (*6*–*8*). MLST has been used previously to study PCP outbreaks, but first-generation sequencing technology (i.e., Sanger sequencing) presents limitations for subpopulations characterization. In the use of Sanger sequencing technology, minor variant detection is generally restricted to strains with a relative abundance of DNA (>20% of the total amount) and requires an amplicon cloning step and the sequencing of >5 clones (*6*,*15*–*17*).

Next-generation sequencing (NGS) technologies yield improved performance. Because of clonal amplification before sequence acquisition, these technologies enable in-depth sequencing and the detection of major, minor, and ultra-minor populations within the same sample (*18*,*19*).

During 2009–2014, the incidence of PCP in all SOT patients in our institution was <1 case/year. In 2014, six cases of PCP were diagnosed among SOT patients over a 3-month period (May–July) and 6 more through August 2015. These grouped cases involved recipients of various organs, 5 heart recipients, 4 kidney recipients, 1 lung recipient, 1 liver recipient, and 1 recipient of both a heart and a kidney. This atypical distribution and the substantial increase of incidence triggered the exploration of this outbreak. In this study, we evaluated the added value of an NGS-MLST strategy in the specific context of a PCP cluster among SOT patients, taking advantage of the specific characteristics of NGS in terms of subpopulation detection.

## Materials and Methods

### Patient Selection and Samples

The Grenoble Alpes University Hospital has a total of 2,200 beds and performs 150 SOTs each year. In 2014, we followed a total of 1,899 SOT patients in our institution (1,380 kidney, 339 liver, 90 heart, and 90 lung recipients). We included 32 PCP patients in the study (Table 1): 12 SOT recipients (mean age 61.6 years, range 34–75 years) and 20 control patients (mean age 57 years, range 7 months–82 years). Control patients were those with sporadic PCP cases diagnosed in the previous 4 years who had diverse underlying conditions and no obvious epidemiologic link. PCP biologic diagnosis was confirmed by quantitative PCR in respiratory samples (bronchoalveolar lavage fluids, bronchoaspiration, or sputum) as described previously (*20*). 

**Table 1:** Main clinical and biologic characteristics of the 12 patients who had received solid organ transplants and 20 control patients in a study of a *Pneumocystis jirovecii* pneumonia outbreak at a university hospital in France, 2009–2015*

Patient	Age, y/sex	Date of diagnosis	Underlying condition	Time since transplant, mo	Sample type	Fungal load, MSG copies/mL	Outcome
T1	34/F	2014 May 20	Pulmonary transplant	60	BAL	1.1 × 10^9^	Died
T2	59/F	2014 May 27	Heart and kidney transplant	300 (H) – 2 (K)	BAL	3.9 × 10^8^	Survived
T3	57/F	2014 Jun 19	Heart transplant	137	BAL	2.7 × 10^8^	Died
T4	65/M	2014 Jul 7	Heart transplant	37	BAL	1.5 × 10^8^	Died
T5	66/M	2014 Aug 19	Kidney transplant	4	Sputum	6.6 × 10^10^	Survived
T6	48/M	2014 Aug 29	Heart transplant	78	BAL	1 × 10^6^	Survived
T7	74/F	2014 Oct 29	Kidney transplant	128	BAL	2 × 10^7^	Died
T8	69/F	2014 Nov 24	Kidney transplant	23	BAL	8.8 × 10^6^	Survived
T9	63/M	2015 Jan 7	Liver transplant	5	BAL	1.1 × 10^4^	Survived
T10	69/M	2015 Jan 10	Kidney transplant	228	Sputum	1.2 × 10^5^	Survived
T11	61/F	2015 Mar 3	Heart transplant	122	BAL	3.1 × 10^3^	Survived
T12	75/F	2015 Aug 26	Heart transplant	95	BAL	2 × 10^5^	Survived
C1	56/M	2012 Aug 25	Liver transplant	6	BA	6 × 10^6^	Died (HCV relapse)
C2	52/M	2013 May 17	Pulmonary transplant	21	BAL	1.7 × 10^5^	Survived
C3	82/F	2012 Mar 9	Rheumatoid polyarthritis	–	BAL	5.6 × 10^3^	Survived
C4	62/M	2013 Aug 29	NHL	–	BAL	4.3 × 10^8^	Survived
C5	66/M	2013 Sep 10	NHL	–	BAL	1.2 × 10^6^	Survived
C6	25/M	2014 Feb 27	HIV	–	BAL	4.2 × 10^8^	Survived
C7	1/F	2014 Apr 9	Primary immunodeficiency	–	BAL	5.2 × 10^9^	Survived
C8	56/M	2014 Apr 19	HIV	–	BAL	4.3 × 10^9^	Survived
C9	47/F	2014 Apr 24	Allografted AML with GVHD	–	BAL	1.4 × 10^6^	Survived
C10	74/M	2014 Jul 1	NHL	–	BAL	6.1 × 10^8^	Survived
C11	77/M	2014 Sep 4	MPN and NHL	–	BAL	1.2 × 10^5^	Survived
C12	82/M	2014 Sep 5	NHL	–	BAL	5.8 × 10^4^	Survived
C13	68/M	2014 Oct 10	Glioblastoma	–	BAL	3.4 × 10^10^	Survived
C14	7 mo/F	2014 Oct 15	Primary immunodeficiency	–	Sputum	4.7 × 10^6^	Survived
C15	75/F	2014 Oct 23	B-chronic lymphoid leukemia	–	BAL	3.4 × 10^5^	Survived
C16	82/F	2014 Dec 5	B-chronic lymphoid leukemia	–	BAL	1.1 × 10^4^	Survived
C17	32/F	2015 Feb 27	Hodgkin's disease	–	BAL	1.2 × 10^4^	Survived
C18	50/M	2015 Feb 28	Hodgkin's disease	–	BAL	6.4 × 10^3^	Survived
C19	78/M	2015 Mar 18	B-chronic lymphoid leukemia	–	BAL	5.9 × 10^6^	Survived
C20	75/M	2015 Mar 22	NHL	–	BA	3.9 × 10^4^	Died
*T1–T12 indicate the 12 solid organ transplant patients from the cluster; C1–C20 indicate the 20 control patients. AML, acute myeloid leukemia; BA, bronchoaspiration; BAL, bronchoalveolar lavage fluids; GVHD, graft-versus-host disease; H, heart; K, kidney; MPN, myeloproliferative neoplasm; MSG, major surface glycoprotein; NA, not applicable; ND, not done; NHL, non-Hodgkin lymphoma; –, nonexistent or unspecified.							

### Clinical Epidemiologic Survey

According to the incubation periods described in the literature (median 53 days, range 1–88 days) (*21*,*22*), we collected data concerning patients’ interactions within the institution from 6 months preceding the date of symptom onset in the first patient until the date of diagnosis of PCP in the last patient. Because colonized patients are potential infectious sources of *P. jirovecii* (*23*,*24*), we considered patients to be possibly contagious throughout the whole incubation period, even in the absence of symptoms and for 3 days after initiating treatment with trimethoprim/sulfamethoxazole. We defined transmission as probable when all of the following criteria were present: 1) the 2 patients were present in the same floor and in the same corridor on the same day, 2) the source patient was in the contagious phase, and 3) the duration from the probable date of transmission to the appearance of the first symptoms was compatible with the incubation period for PCP. If 1 of these criteria was missing, transmission was defined as possible. We defined the incubation period for PCP as the time elapsed from the strain transmission to the onset of symptoms.

### MLST Strategy

We used an MLST strategy to genotype *P. jirovecii* strains, as recently recommended (*25*). We chose a combination of 3 loci belonging to the superoxide dismutase (*SOD*), the mitochondrial large subunit of ribosomal RNA (*mtLSU*), and the cytochrome b (*CYTB*) genes. This scheme displays a high level of strain discrimination and is adapted for accurate *P. jirovecii* strain typing (*14*). We designed new primers for each locus to obtain ≈750 bp amplicons depending on the chosen NGS procedure, which was performed by using GS Junior + (Roche Diagnostics, Meylan, France). Because we used extended sequences to characterize our strains, we could not refer to any previously used sequence type labeling. Thus, we arbitrarily named haplotypes with a capital letter (A), a number (1), and a lowercase letter (a) corresponding to *mtLSU*, *CYTB*, and *SOD* loci. We then labeled final genotypes by the association of the 3 different haplotypes (e.g., A1a). Primers, PCR conditions, and haplotype sequences are summarized in Technical Appendix Tables 1.

### Amplicon Library Preparation and Emulsion PCR

We extracted total DNA by using the QIAmp DNA Mini Kit (QIAGEN, Hilden, Germany), according to the manufacturer’s instructions and as previously described (*20*), and stored extracts at –20°C until library preparation. We prepared the amplicon library by using the universal tailed amplicon sequencing design. We first amplified each region of interest by using specific oligonucleotides coupled to M13 universal primers (PCR1) and then targeted these universal sequences in a second PCR (PCR2) to add a multiplex identifier and the sequencing primers as described in the Guidelines for Amplicon Experimental Design—454 Sequencing System (Roche Diagnostics). We purified PCR products with magnetic beads from an AMPureXP Kit (Beckmann Coulter, Beverly, MA, USA). After qualitative and quantitative analysis, we diluted purified PCR2 products to 1 × 10^9^ molecules/mL and pooled an equal volume of each dilution to generate the amplicon library. We performed emulsion PCR as recommended in the GS Junior + library preparation procedure.

### NGS and Limit of Detection

We performed NGS to sequence 700–800 bp fragments, according to the manufacturer’s instructions, using a forward and reverse sequencing strategy for better coverage. Ninety-six PCR products were distributed in 2 comparably filled runs. We added a control sample to assess the limit of detection of subpopulations under our experimental conditions. This control consisted of 3 purified *mtLSU* amplicons belonging to 3 unique haplotypes, based on the results of the first run at 3 different ratios: 98.9%, 1.0%, and 0.1%.

### Bioinformatic Analysis

We analyzed NGS results by using Amplicon Variant Analyzer and GS Reference Mapper software (Roche Diagnostics) and compared sequences to their respective reference sequences; GenBank accession nos. were NC_020331.1 (*mtLSU*), AF146753.1 (*SOD*), and AF320344.1 (*CYTB*). Each polymorphism position was verified and visually validated. We applied a 1% threshold for minor variant consideration based on the result of our artificial mix sample. We calculated haplotype frequency by using Amplicon Variant Analyzer software (dividing number of reads corresponding to the haplotype by the total number of reads for 1 locus).

### Statistical Analysis and Hunter Index

We compared co-infection proportions between cluster and control populations by using the Fisher exact test, calculated a p value, and defined statistical significance as p<0.05. The Hunter index (D) was calculated to evaluate the discriminatory power of the 3 loci and their association, as previously described (*26*). Only single strains or strains with an easy extrapolation of the major genotype (variants in only 1 locus or presence of ultra-major variants in all loci) were taken into account for the D calculation. Related samples sharing the outbreak genotype were also excluded, except 1.

## Results

### NGS Performance

The mean sequencing depths for NGS of *P. jirovecii* strains were 810× (5×–1,998×, median 739×) and 265× (8×–1,268×, median 233×) for the first and second runs, respectively. Sequence quality was assessed using FastQC software (http://www.bioinformatics.babraham.ac.uk). For the first run, the median Phred score (or Qscore) per base, up to a 750 bp read length, was >24 (corresponding to a probability of error <0.4%); for the second run, the median Phred score was >20 until 400 bp read length (corresponding to a probability of error <1%).

### Limit of Detection

In the artificial control sample consisting of 3 *mtLSU* haplotypes at defined concentrations (C, 98.9%; B, 1%; and F, 0.1%), the C and B haplotypes were correctly detected with NGS at 99% (C) and 1% (B). The ultra-minor F genotype was not detected, a finding consistent with the limited analysis depth obtained for this sequence (220×).

### Nucleotide Polymorphisms, Haplotypes, and Genotypes Determination

We genotyped 12 *P. jirovecii* samples from the SOT patient cluster and 20 samples from unrelated control patients by using the 3 loci NGS-MLST strategy. Analysis of extended 732 bp *mtLSU* sequences revealed 4 new polymorphisms located both upstream and downstream of the amplicons classically used for genotyping (Technical Appendix Figure). These polymorphisms correspond to 3 single-nucleotide polymorphisms at positions 13002, 13505, and 13543 in the mitochondrial genome (GenBank accession no. NC_020331.1) and 1 multinucleotide polymorphism between 13554 and 13560. By contrast, extended *CYTB* and *SOD* amplicons did not show new polymorphisms.

Among the 32 samples, we identified 22 different haplotypes for *mtLSU*, 14 for *CYTB*, and 4 for *SOD*, with major and minor variants being considered (Table 2). We identified C2a as the major genotype in 5 SOT patients from the cluster: 1 double (heart and kidney) recipient (T2), 1 heart recipient (T4), and 3 renal transplant recipients (T5, T8, and T10). We also detected this C2a genotype as a minor variant (2%) in 1 other SOT patient belonging to the 2014–2015 outbreak cluster (T7). With major and minor genotypes being considered, 6/12 SOT patients were carrying the C2a genotype. We did not detect this unusual genotype in any control patient, either as a major or minor strain (Table 2).

**Table 2:** Results of next-generation multilocus sequence typing for the 3 targeted loci of *Pneumocystis jirovecii* strains in a study of a *P. jirovecii* pneumonia outbreak at a university hospital in France, 2014–2015*

Patient	*mtLSU*				*CYTB*				*SOD*			Final major genotype
	No. reads	No. haplotypes	Major haplotype		No. reads	No. haplotypes	Major haplotype		No. reads	No. haplotypes	Major haplotype	
T1	1,413	1	B		531	1	1		946	1	A	B1a
T2	1,062	1	C		1,361	1	2		2,506	1	A	C2a
T3	638	4	N		84	1	3		491	1	B	N3b
T4	87	1	C		215	1	2		204	1	A	C2a
T5	460	1	C		1,030	1	2		337	1	A	C2a
T6	42	1	E		237	1	2		171	1	A	E2a
T7	546	1	C		1,088	1	2		303	4	B	C2b†
T8	606	2	C		1,136	2	2		808	1	A	C2a
T9	194	1	C		160	1	2		8	1	B	C2b
T10	415	1	C		1,360	1	2		898	1	A	C2a
T11	420	3	O		370	2	1		298	3	B	O1b
T12	658	9	F		809	4	3		781	1	A	F3a
C1	318	3	P		1,046	1	3		1,268	1	A	P3a
C2	422	8	B		339	4	3		364	2	A	ND
C3	350	1	I		564	1	3		1,268	1	B	I3b
C4	292	2	N		492	1	3		427	1	B	N3b
C5	71	1	B		651	1	2		880	1	B	B2b
C6	368	12	P		473	3	3		575	4	B	ND
C7	153	2	F		103	2	3		54	4	A	F3a
C8	383	5	I		697	2	3		653	4	B	ND
C9	148	2	F		104	4	2		154	1	a	F2a
C10	934	2	F		1,146	5	2		550	3	a	F2a
C11	229	4	B		750	3	3		119	2	b	B3b
C12	580	2	G		398	1	1		509	1	a	G1a
C13	869	1	F		1,998	1	3		663	1	a	F3a
C14	901	9	B		1,403	1	2		411	1	b	B2b
C15	653	1	N		1,664	2	3		5	1	a	N3a
C16	1,275	1	F		1,052	1	11		591	1	a	F11a
C17	372	10	P		434	3	2		361	1	b	ND
C18	296	1	F		271	1	3		15	3	b	F3b
C19	948	2	F		795	4	1		494	1	a	ND
C20	243	5	A		284	9	2		240	2	b	ND

*T1–T12 indicate the 12 solid organ transplant patients from the cluster; C1–C20 indicate the 20 control patients. Underlined genotypes were included for Hunter Index calculation. *CYTB*, cytochrome b; *mtLSU*, mitochondrial large subunit of ribosomal RNA; ND, undetermined genotypes when haplotypes could not be cross-linked (multiple variants in >1 locus without any clear predominance); *SOD*, superoxide dismutase. †Patient carrying the C2a genotype as a minor strain.

### Epidemiologic Investigation

**Figure 1:**
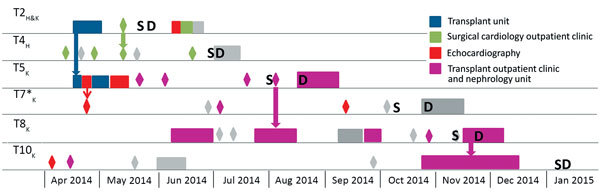
Transmission map illustrating space and time events for cluster patients sharing the clonal C2a genotype identified in a study of a *Pneumocystis jirovecii* pneumonia outbreak at a university hospital in France, 2009–2015. Rectangles indicate hospitalization periods of >1 day; diamonds indicate 1-day presence in the institution (e.g., emergency services, imaging, laboratory, outpatient clinics). Places involved in the transmission network are shown in color, whereas units not involved are shown in gray. Thick arrow refers to probable nosocomial transmission of *P. jirovecii* between 2 patients who were present in the same place (same floor and same corridor) on the same day. Thin arrow represents possible nosocomial transmission of *P. jirovecii* between T5 and T7 (patients were in perpendicular corridors). Subscript letters on patient labels indicate type of transplant (H, heart; K, kidney). D, date of diagnosis and treatment initiation; S, date of symptoms onset. Asterisk indicates that patient was carrying the C2a genotype as a minor strain.

We noted the transmission networks between the 6 SOT patients infected with the C2a strain, the time to diagnosis, and the incubation for PCP (Table 3). The transmission map (Figure 1) pointed out potential nosocomial transmission of the C2a strain involving both heart and kidney transplant units, with the index patient being patient T2, who was a long-standing heart recipient who received a kidney transplant 2 months before PCP diagnosis. Transmission was probable in the following patients: between T2 and T5, within the nephrology department; between T2 and T4, within the heart surgery outpatient clinic; between T5 and T8, in the nephrology department; and between T8 and T10, in the transplant outpatient clinic. Transmission between T5 and T7 was considered only possible because the patients were on the same floor the same day but in perpendicular corridors, and the incubation time for patient T5 was relatively short (4 days). Of note, 4 of the 5 suspected nosocomial transmissions of this strain occurred in outpatient clinic areas (T2/T4, T5/T7, T5/T8, and T8/T10), and 1 occurred in the transplant unit (T2/T5). According to the transmission map, PCP incubation intervals (time elapsed between strain acquisition and symptom onset) were 48 (T10), 49 (T4), 99 (T8), 109 (T5), and 170 (T7) days.

**Table 3:** Epidemiologic information on patients found to be carrying the C2a genotype as a major or a minor strain in a study of a *Pneumocystis jirovecii* pneumonia outbreak at a university hospital in France, 2014–2015

Patient	Transmission			Date of symptom onset	Date of diagnosis	Time to diagnosis, d	Incubation, d
	Source	Date	Place				
T2	Index patient	ND	ND	2014 May 25	2014 May 30	5	ND
T4	T2	12 May 2014	Surgical cardiology outpatient unit	2014 Jul 01	2014 Jul 06	5	49
T5	T2	2014 Apr 18	Kidney transplant unit	2014 Aug 05	2014 Aug 19	14	109
T7†	T5	2014 Apr 22	Cardiac echography unit	2014 Oct 09	2014 Oct 29	20	170
T8	T5	2014 Aug 07	Transplant consultation unit	2014 Nov 14	2014 Nov 24	11	99
T10	T8	2014 Nov 23	Transplant consultation unit	2015 Jan 05	2015 Jan 10	5	48

*Date of transmission based on transmission map and previously defined criteria of transmission. ND, not determined. †Patient carrying the C2a genotype as a minor strain.

### Variants Analysis and Mixed Infections

**Figure 2:**
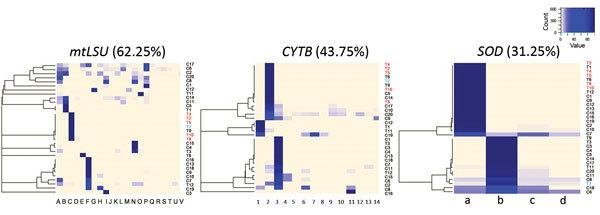
Heatmaps representing distribution for *mtLSU*, *CYTB*, and *SOD* variants among patients infected during a *Pneumocystis jirovecii* pneumonia outbreak at a university hospital in France, 2014–2015. Patients shown in red correspond to cluster patients sharing the common C2a genotype. Patient shown in blue is the patient having the C2a genotype as minor variant (2%). Percentages shown indicate the level of mixed-strain infections for each locus.

Among the 32 respiratory samples, 11 (34.4%) contained a single and unique *P. jirovecii* strain with no minor variants detected. Thus, 21/32 (65.6%) respiratory samples consisted of multiple *P. jirovecii* strains, and 17/32 (53.1%) had >2 different *P. jirovecii* strains. Co-infection proportions were significantly different between the cluster group (5/12 [41.7%] mixed infections) and the control group (17/20 [85.0%] mixed infections; p<0.05). The exact number of strains within each sample was sometimes difficult to determine because minor haplotypes could not always be associated. However, we observed an average minimum of 5 strains per sample (corresponding to the highest number of alleles in 1 locus), ranging from 2 to 12, in these co-infected patients. In more than half of the patients, we detected minor variants with a proportion <10%; 18/32 patients (56.2%) had >1 subpopulation <10%, and 9/32 (28.1%) had only minor variants <10%. The *mtLSU*, *CYTB*, and *SOD* loci enabled detection of 62.5% (20/32), 43.75% (14/32), and 31.25% (10/32) mixed strain infections, respectively. We plotted heatmaps representing strain distribution patterns for each locus (Figure 2). The co-infection proportions were comparable between the 2 sequencing runs (61% and 65%).

### Discriminatory Index of the Extended Amplicons

We calculated the Hunter index (D) for each locus of 19 strains (Technical Appendix Table 3), excluding the clonal C2a strains and uninterpretable genotypes (*26*). The discriminatory index of the *mtLSU* locus D was 0.754 when considering only known polymorphisms and 0.883 when considering both known and newly described polymorphisms. Similarly, in terms of *CYTB* and *mtLSU* loci association, D increased from 0.918 with only known polymorphisms to 0.953 when adding the newly described polymorphisms. When the 3 loci were considered, D further increased from 0.971 to 0.982.

## Discussion

An outbreak of 12 diagnosed PCP cases occurred among SOT recipients in our institute during 2014–2015. This cluster consisted of patients carrying different transplanted organs who were followed up in different organ specialty units. We used an exhaustive NGS-MLST approach to accurately characterize the major and minor strains implicated.

To our knowledge, PCP outbreaks among SOT patients have been commonly described in renal transplant patients, reported more rarely in hepatic transplant recipients, and never reported in cardiac transplant patients (although sporadic cases exist in this population). Also, most reported grouped cases of PCP in SOT recipients described cases occurring in patients grafted with the same organ; grouped cases in patients transplanted with different organs rarely have been described (*4*,*5*). In our study, even though the SOT cluster patients were followed up by different units, they belonged to the same epidemic event according to genotyping results. Five SOT cluster patients shared a common major genotype, and the transmission map (Figure 1) pointed out potential nosocomial transmission of this strain, which might have been present in both the cardiology and nephrology transplant units. This genotype was not detected in the control group, confirming that this *P. jirovecii* strain is not commonly present within our institute. 

The epidemiologic survey, guided by the genotyping results, identified potential dates and places of transmission between the patients infected with the same *P. jirovecii* strain. Five probable and 1 possible transmission events between SOT patients were revealed. Of note, 4/5 suspected transmission events occurred in outpatient areas. Waiting rooms and day hospital corridors have been thought to promote person-to-person contact and increase the risk for *P. jirovecii* transmission (*7*). The wearing of masks in waiting rooms frequently visited by immunocompromised patients was recently suggested for patients with respiratory symptoms but should also be recommended to any patient, given that asymptomatic carrier state or patients in incubation state might also be at risk for transmission (*7*,*27*). Also, patients could have met in certain common areas of the hospital (e.g., cafeteria and corridors), encounters that are usually not in the medical records. Recent studies confirmed the high probability of *P. jirovecii* airborne transmission or transmission between patients and healthcare workers (*23*,*28*). In the affected units, it would have been of interest to screen healthcare workers or air samples to identify asymptomatic carriers of the C2a *P. jirovecii* strain or its presence in the environment.

In our study, we used a 1% threshold for minor variant detection according to our artificial mix control. By using this threshold, we detected 21/32 (66%) cases with mixed-strain infections and 17/32 (53%) cases with >2 strains. Previous genotyping studies described *P. jirovecii* multiple infections with diverse proportions of different strains (from 20% to 75%) (*29*–*31*). Hauser et al. (*29*) and Gits-Muselli et al. (*12*) described mixed populations in 70% of cases with the use of PCR-single-strand-conformation polymorphism or microsatellite analysis. Our proportion of mixed strains was even higher (85%) when we considered only the control patients, which might more accurately reflect the reality of *P. jirovecii* strain diversity in a nonoutbreak environment. Ultra-deep sequencing of *P. jirovecii* strains outside an outbreak context has indicated a 92% proportion of coinfections (*32*). The proportion we observed with infections involving >2 strains is higher than previously described (*30*) because of the more accurate variant detection <10% provided by NGS. We noted a difference in the proportion of co-infections between the cluster group (42%) and control group (85%) (p<0.01) despite identical mean sequencing depth. We can hypothesize that the C2a strain has a particular intrinsic virulence or that this cluster has some specific characteristic, but further study would be required.

By using NGS, we found 1 confirmed C2a genotype as the minor strain in patients belonging to the cluster. The ratio among the different *P. jirovecii* strains can vary over time in a given patient (*33*). Thus, a previous minor strain can theoretically become a major strain over time. The patient with a minor C2a strain (T7) was thus added to the transmission map. NGS also enabled us to exclude other SOT and control patients from the transmission map with a higher degree of certainty compared with first-generation sequencing (Sanger sequencing), which is not accurate for minor strain detection <20%. Therefore, NGS-MLST appears to have a higher accuracy for describing an outbreak event and for specifying the transmission network.

For experimental purposes, we designed new primers to generate longer amplicons. We discovered 4 new mutations within the 732 bp *mtLSU* amplicon but no new polymorphism for the *SOD* or *CYTB* loci. Currently, no established MLST scheme for PCP typing exists; the 3-loci (*mtLSU*, *CYTB*, and *SOD*) scheme has been proposed and previously used in the a context of a PCP outbreak (*3*,*14*). The 5th European Conference on Infections in Leukemia also recommended this MLST scheme for *P. jirovecii* typing (*25*). The internal transcribed spacer regions have been used but are difficult to amplify and are at risk for in vitro recombination (*14*,*34*). Extended 732 bp *mtLSU* and *CYTB* sequence polymorphisms conferred a high discriminatory power (D>0.95), considered to be sufficient for strain discrimination (*35*). In addition, *mtLSU* and *CYTB* amplification is facilitated because of their mitochondrial origin (multicopy genes). Hence, the lengthened 732 bp *mtLSU* PCR product could be of interest in light of the previously described 300 or 370 bp amplicons (*14*). The sequencing of 732 bp *mtLSU* and *CYTB* could be proposed as a rapid screening method to evaluate strain convergence, whereas *SOD* or internal transcribed spacer regions could be sequenced in a second step. Further studies will be needed to assess the added value of these newly described polymorphisms.

Although our study revealed new features in *P. jirovecii* typing, it did not provide evidence for or against features such as diploidy or mitochondrial heteroplasmy of *P. jirovecii* during PCP (*32*,*36*). None of our 32 samples exhibited a clear 50/50 allele repartition, which would have suggested heterozygosity, but the presence of heterozygote stages among the described genotypes cannot be entirely ruled out. The mitochondrial genes *mtLSU* and to a lesser extent *CYTB*, displayed higher diversity than the nuclear locus *SOD.* However, this finding is not sufficient to confirm mitochondrial heteroplasmy because these differences might also have been attributable to the higher rate of polymorphism in the mitochondrial loci. One third of the samples displayed a unique genotype, suggesting that mitochondrial heteroplasmy was not present during these PCP infections.

This study demonstrated the clear added value of NGS-MLST to analyze major and minor strains in epidemiologic *P. jirovecii* studies. NGS is increasingly attractive because of the rapid development of bioinformatics and the reduced cost of this approach (*37*). We believe that NGS-MLST represents the next generation of MLST and that this method will become the new standard for strain typing in the next decade, especially for microorganisms that are not cultivable in vitro.

Technical Appendix Additional description of primers, PCR conditions, haplotype sequences, and new polymorphisms located both upstream and downstream of the amplicons classically used for genotyping in a study of a *Pneumocystis jirovecii* pneumonia outbreak at a university hospital in France, 2009–2015.
