# Supplementary material for: Added Value of Next-Generation Sequencing for Multilocus Sequence Typing Analysis of a Pneumocystis jirovecii Pneumonia Outbreak
Source: Emerg Infect Dis. 2017 Aug;23(8):1237–45. doi: 10.3201/eid2308.161295 (PMC5547796; doi:10.3201/eid2308.161295)
Supplement: Technical Appendix — Additional description of primers, PCR conditions, haplotype sequences, and new polymorphisms located both upstream and downstream of the amplicons classically used for genotyping in a study of a Pneumocystis jirovecii pneumonia outbreak at a university hospital in France, 2009–2015. [file 16-1295-Techapp-s1.pdf]

Article DOI: https://doi.org/10.3201/eid2308.161295

Added Value of Next-Generation Sequencing for Multilocus Sequence Typing Analysis of a *Pneumocystis jirovecii* Pneumonia Outbreak

Technical Appendix

Technical Appendix Table 1. PCR1 and PCR2 primers and amplification conditions*

Locus	Primers	Amplicon size, bp	Annealing	Mg2+, nM	No. cycles
PCR 1 (specific amplification)					
mtLSU	F: Universal tail-TTTTGCATAATGGGTCAGCA;<br>R: Universal tail-TCGGCGAATAGGATTTTCAC	732	65°C	1.5	35
CYTB	F: Universal tail-CTCCCAGAATTCTCGTTTGG<br>R: Universal tail-GTCCAAGCGTCACAAAAGGT	735	65°C	1.5	35
SOD	F: Universal tail-CTTTCTCATGATTTGCTTGAGG<br>R: Universal tail-CCTTTTGCTTCATTAGTCAAAAAATTA	761	60°C	2.5	40
PCR 2 (universal amplification)					
mtLSU<br>*CYTB*<br>*SOD*	F: Primer(A)-Key-MID-Universal tail<br>R: Primer(B)-Key-MID-Universal tail	≈800	65°C	3	40

*Universal tail = M13 universal primer (Forward: TGTAAAACGACGGCCAGT; Reverse: CAGGAAACAGCTATGACC); mtLSU = mitochondrial rRNA large-sub-unit; CYTB = cytochrome B; SOD: superoxide dismutase.

Technical Appendix Table 2. Polymorphisms association corresponding to each haplotype for *mtLSU*, *CYTB*, and *SOD* loci*

mtLSU

	Polymorphisms									
Haplotype	13002	13215	13378	13505	13543	13554	13556	13557	13558	13560
A	C	C	C	C	C	C	G	T	A	T
B	C	C	T	C	C	C	G	T	A	T
C	C	C	T	C	C	A	T	A	C	G
D	C	C	C	C	C	A	T	A	C	G
E	C	T	C	C	T	C	G	T	A	T
F	C	A	C	C	C	C	G	T	A	T
G	C	A	C	T	T	C	G	T	A	T
H	C	A	T	C	C	C	G	T	A	T
I	G	T	C	C	C	C	G	T	A	T
J	G	T	T	C	C	C	G	T	A	T
K	G	C	T	C	C	C	G	T	A	T
L	C	T	T	C	C	C	G	T	A	T
M	C	A	C	C	T	C	G	T	A	T
N	G	C	C	C	C	C	G	T	A	T
O	G	A	C	C	C	C	G	T	A	T
P	C	C	T	C	C	C	G	T	A	T
Q	C	C	C	C	T	C	G	T	A	T
R	C	C	T	C	T	A	T	A	C	G
S	C	T	C	C	T	A	T	A	C	G
T	C	C	T	C	T	C	G	T	A	T
U	C	A	C	C	C	A	T	A	C	G
V	G	A	T	C	C	C	G	T	A	T

CYTB

	Polymorphisms												
Haplotype	279	299	348	362	369	517	547	566	675	742	832	833	838
1	T	C	A	C	G	T	C	C	A	C	T	T	C
2	C	C	A	C	G	C	C	C	A	C	T	T	T
3	C	C	A	C	G	C	C	C	A	C	T	T	C

4	C	C	A	C	G	C	C	T	A	C	T	T	C
5	C	C	G	C	G	C	C	C	A	C	T	T	C
6	C	C	A	C	G	T	C	C	A	C	T	T	C
7	C	C	G	C	G	T	C	C	A	C	T	T	C
8	T	C	G	C	G	T	C	C	A	C	T	C	C
9	C	C	A	C	G	T	C	C	A	C	T	T	T
10	C	C	G	C	G	C	C	C	A	C	T	T	T
11	T	C	A	C	G	C	C	C	A	C	T	T	C
12	C	C	G	C	G	T	C	C	A	C	T	T	T
13	T	C	A	C	G	C	C	C	A	C	T	T	T
14	C	C	A	C	G	C	C	T	A	C	T	T	T

SOD

	Polymorphisms		
Haplotype	110	179	215
a	C	T	T
b	T	T	C
c	T	T	T
d	C	T	C

*Polymorphism positions are indicated, according to their respective GenBank reference sequence: NC_020331.1 (*mtLSU*), AF146753.1 (*SOD*), AF320344.1 (*CYTB*).

Technical Appendix Table 3. Hunter Index: (D) value for *mtLSU*, *CYTB,* and *SOD* loci independently and in association, with previously described (classical) amplicons and new (extended) amplicons on 19 unrelated strains presenting a very major genotype

Amplicon type	*mtLSU*	*CYTB*	*SOD*	*mtLSU-CYTB*	*mtLSU-CYTB-SOD*
Classical amplicons (*1*)	4 genotypes<br>D = 0.754	4 genotypes<br>D = 0.684	2 genotypes<br>D = 0.526	10 genotypes<br>D = 0.918	15 genotypes<br>D = 0.971
Maitte et al. (*1*)	D = 0.751	D = 0.794	D = 0.57	D = 0.957	D = 0.987
New amplicons	9 genotypes<br>D = 0.883	4 genotypes<br>D = 0.684	2 genotypes<br>D = 0.526	13 genotypes<br>D = 0.953	16 genotypes<br>D = 0.982

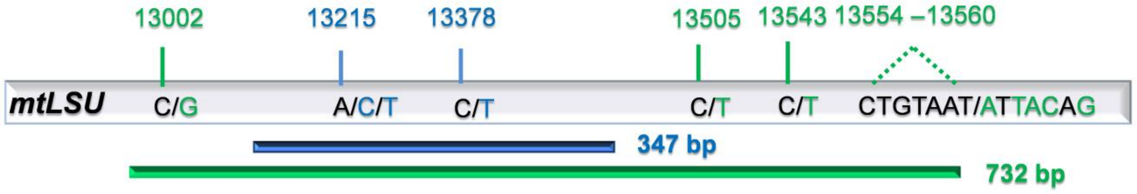

Technical Appendix Figure. New polymorphisms described on the *mtLSU* extended locus. Blue line shows 347 bp sequence corresponding to the amplification with previously described primers (*1*). Green line shows 732 bp sequence corresponding to the amplification using primers described in this study. Bars show single nucleotide or multinucleotide polymorphisms with their corresponding positions on the mitochondrial genome. Blue-colored polymorphisms are previously described, whereas green-colored polymorphisms are newly described in this study.

Reference

1. Maitte C, Leterrier M, Le Pape P, Miegeville M, Morio F. Multilocus sequence typing of *Pneumocystis jirovecii* from clinical samples: how many and which loci should be used? J Clin Microbiol. 2013;51:2843–9. PubMed http://dx.doi.org/10.1128/JCM.01073-13
